# Recommendations on the management of meningioma and sex hormone therapy: The results of a collaborative effort between neurosurgical, endocrine and gynecological societies

**DOI:** 10.1016/j.bas.2024.104154

**Published:** 2024-12-09

**Authors:** Gilles Reuter, Iulia Potorac, Carlien de Herdt, Linda Ameryckx, Géraldine Brichant, Sébastien Froelich, Bertrand Baussart, Steven De Vleeschouwer, Tomas Menovsky, Tony Van Havenberghe, Patrice Finet, Michael Bruneau, Axelle Pintiaux

**Affiliations:** Department of Neurosurgery, CHU de Liege, Liège, Belgium; Department of Endocrinology, CHU de Liege, Liège, Belgium; Department of Endocrinology, UZ Antwerpen, Antwerpen, Belgium; Department of Gynecology, UZ Antwerpen, Antwerpen, Belgium; Department of Gynecology, CHU de Liege, Liège, Belgium; Department of Neurosurgery, Hopital Lariboisiere, Paris, France; Department of Neurosurgery, UZ Leuven, Leuven, Belgium; Department of Neurosurgery, UZ Antwerpen, Antwerpen, Belgium; Department of Neurosurgery, ZAS, Antwerpen, Belgium; Department of Neurosurgery, UCL Saint Luc, Brussels, Belgium; Department of Neurosurgery, UZ Brussels, Brussels, Belgium; Department of Gynecology, CHU de Liege, Liège, Belgium

**Keywords:** Meningioma, Progestin, Oral contraceptive, Menopause, Sex hormone therapy

## Abstract

**Introduction:**

Exogenous and endogenous sex hormones, especially Progesterone agonists, may be causally linked to meningioma progression. Cessation of treatment leads to stabilization or regression of Progestin-induced meningioma. In many cases, avoiding sex hormone therapy may be possible in the context of meningioma treatment. However, hormonal treatment is not always easily replaceable and concise real-world recommendations regarding sex hormones and meningioma are lacking.

**Material and methods:**

A combined effort was initiated between Neurosurgical, Gynaecological and Endocrinological societies of Belgium to gather relevant information regarding sex hormone therapies and meningioma. After complete literature review, consensual recommendations were established.

**Results:**

Collegial recommendations regarding sex hormones therapies and meningioma in the context of oral contraceptives, menopause hormonal treatment, fertility treatment, pregnancy and gender-affirming therapies are emitted and nuanced.

**Discussion and conclusion:**

Withdrawal and monitoring of sex hormone therapies are discussed in detail.

A decision tree regarding Meningioma and Combined contraception, Progestin Contraception, Menopause Hormonal treatment, Progestin and Gender-affirming therapy is suggested.

## 1 Introduction

Meningiomas, originating from the arachnoid gap cells in the arachnoid surrounding the brain and spinal cord, are the most commonly reported primary brain tumors (Rogers et al., 2015). The annual incidence is approximately 6–8 cases per 100,000, with a peak occurrence in middle-aged patients and a female-to-male ratio ranging from 2 to 3.5:1, depending on the age group (Ostrom et al., 2018; Baldi et al., 2011; Klaeboe et al., 2005). Interestingly, incidental meningiomas are frequent and can be found with a prevalence of 0.9% (Vernooij et al., 2007). Notably, in rare prepubertal meningiomas, the female-to-male ratio may be inverted, while the peak female-to-male ratio is most pronounced among patients aged 35–44 years (Wiemels et al., 2010b).

The vast majority of these tumors are classified as benign (World Health Organization [WHO] grade 1). Still, approximately 15%–20% display atypical behavior (WHO grade 2), and in rare instances (1%–2% of cases), meningiomas can be malignant (WHO grade 3) (Louis et al., 2016).

Several factors have been involved in the formation of meningiomas, with neurofibromatosis type 2 and ionizing radiation being well-established contributors (Wiemels et al., 2010b). Sex hormones have been suspected in the pathogenesis of meningiomas because of higher incidence among women, (Klaeboe et al., 2005; Wiemels et al., 2010a), accelerated growth during pregnancy (Kerschbaumer et al., 2015; Lusis et al., 2012; Dumitrescu et al., 2014), increased risk in patients with breast cancer (Lopez-Rivera et al., 2020; Miller, 1986), presence of hormone receptors in meningiomas (Agopiantz et al., 2023) and increased risk of meningioma in men treated with antiandrogens (Weill et al., 2021). These associations raise questions regarding the safety of sex hormone therapy in the management of meningiomas.

In many cases, avoiding sex hormone therapy may be possible in the context of meningioma treatment. However, there are instances when hormonal treatment is not easily replaceable. For example, certain progestins such as Cyproterone Acetate (CPA), very effective in the treatment of severe hyperandrogenism, and Nomegestrol Acetate (NA), a valid alternative to hysterectomy in specific conditions, have been associated with the progression of meningioma (Weill et al., 2021; Hoisnard et al., 2022).

As a result, it is crucial for every case of meningioma associated with any sex hormone treatment to be the subject of a comprehensive discussion between the patient, the neurosurgeon and the patient's gynecologist and/or endocrinologist. Consequently, the current challenge is to provide patients with meningioma with straightforward and reliable advice regarding their hormonal treatment based on the current scientific data. Previous studies summarized the role of sex hormones and meningioma (Hage et al., 2021).

The purpose of this article is to update the literature review and to provide guidance to physicians in counseling their patients with meningiomas and concurrent hormonal treatment.

## 2 Methods for literature review

We conducted a research for this review in Pubmed/Medline, Scopus and the NIH clinical trials database, reporting our findings according to the Preferred Reporting Items for Systematic Reviews and Meta-Analysis (PRISMA) statement standards (Moher et al., 2009, 2010).

We performed our search for English articles published until the 1st of April 2024. The following terms were used to search for titles and abstracts using the Boolean operator “and”: “Meningioma” and “Sex Hormone”, “Meningioma” and “Progestin”, “Meningioma” and “Progesterone”, “Meningioma” and “Estrogen”, “Meningioma” and “Androgen”. We solely selected studies that evaluated meningioma and sex hormone therapies. The articles were all managed with Endnote X9 (Thompson Reuters, Carlsbad, California, USA). The initial search delivered 1183 articles. We removed all duplicates (n = 777) and excluded all non-English articles (n = 17). Articles were considered non-relevant when meningioma and sex hormones was not the main topic of the article. After removing non-relevant and non-retrieved (i.e. full text unavailable) articles (n = 156), 237 articles were ultimately evaluated. Additional reports were included when relevant, identified through cross-reference checks.

We then suggested consensual recommendations edited by neurosurgeons, gynecologists and endocrinologists who take part in Belgian national scientific societies: *the skull base and vascular section* of the Belgian Society for Neurosurgery (bsn.be) and the Royal College of French-Speaking Belgian Obstetricians and Gynecologists (CRGOLFB Collège Royal des Gynécologues et Obstétriciens de langue française de Belgique) and the Belgian Menopause Society (BMS). This article is written on behalf of these scientific societies.

### 2.1 Risk factors associated with meningiomas

**Genetic Disorders (NF2, NF1, PTCH1, CREBBP, VHL, PTEN, CDKN2A):** Individuals with known genetic disorders such as neurofibromatosis type 2 (NF2), neurofibromatosis type 1 (NF1), *PTCH1* mutations*, CREBBP* mutations, von Hippel-Lindau (VHL) syndrome, *PTEN* mutations, Goltz-Gorlin syndrome (naevoid basal cell carcinoma syndrome), Cowden syndrome, Multiple Endocrine Neoplasia type 1 (MEN1), Werner Syndrome, Rubinstein-Taybi, *BAP1* tumor predisposition, *CDKN2A* mutations or familial meningiomatosis (germline mutations of *SMARCB1* and *SMARCE1* genes) are at the highest risk of developing meningiomas. The spectrum of genetic anomalies predisposing patients to the development of meningiomas continues to expand (Kerr et al., 2018).

**Personal and/or Familial History of Meningioma or Breast Cancer:** Having a personal history of meningioma or a family history of meningioma (odds ratio [OR] 4.4, 95% CI 1.6–11.5) (Claus et al., 2011) or breast cancer ([OR], 1.41; 95% CI, 0.99–2.02) can increase an individual's risk (Rao et al., 2009). Vice versa, having a meningioma increases the risk of having a breast cancer ([OR], 9.87; 95% CI, 7.31–13.32) (Degeneffe et al., 2023). Demonstrating their intricate relationship, there are also instances of tumor-to-meningioma metastasis, where meningiomas serve as host target for metastatic spread of breast cancers (Caroli et al., 2006; Lee et al., 1998; Turner et al., 2021).

**History of Ionizing Radiation:** Exposure to ionizing radiation, especially at a young age, is a well-established risk factor for meningioma. This factor is strongly associated with increased risk (Umansky et al., 2008). In a series of children cranially irradiated for leukemia, 22% developed at least 1 meningioma (mean, 25 years after irradiation; range, 14–34 years) (Banerjee et al., 2009). Compared to spontaneous meningioma, there is a higher incidence of grade 2 and 3 meningioma, there is no female predominance and there is a higher risk of multiple meningioma. The latency period is depending from the dose of radiation and combination with chemotherapy (Yamanaka et al., 2017).

**Race (Black Race):** Some studies have shown variations in meningioma risk among different racial and ethnic groups, with higher rates in certain populations, including individuals of Black race (Dolecek et al., 2015).

**Obesity:** There is emerging evidence suggesting that obesity is associated with an increased risk of meningioma, although the association is not as strong as the factors mentioned above. The exact mechanism is unknown, but might be related to high levels of free insulin-like growth factor 1 (Shao et al., 2014). Obese males are more likely to have meningiomas of the skull base compared with other locations, but this association was not found in females (Khazanchi et al., 2024a, 2024b).

**Age (Older Patients):** The risk of meningioma follows a linear increase with a higher incidence in older individuals (incidence of 50 per 100,000 patients older than 85) (Wiemels et al., 2010a). A major cause of this increased incidence is the increase in the number of incidentally diagnosed meningioma.

**Head trauma:** Epidemiological studies have suggested a correlational relationship between traumatic brain injury and meningiomas with reports of cases who developed meningioma at the site of prior head trauma (Francois et al., 2010). However, the most recent literature reviews on the subject found no association between head injury and meningiomas (Shah et al., 2022).

The influence of sex hormones.

The influence of sex hormones on meningiomas has been recognized since the late 1920s, initially described by Cushing and Eisenhardt in a patient with rapid symptom progression during pregnancy (Cushing, 1929).

The potential impact of sex hormones on meningiomas is further suggested by various observations:

**Higher Incidence Among Women**: Meningiomas occur more frequently in women, predominantly in elderly women. According to several epidemiological studies, in the age group of 20–34, the observed sex ratio (female:male) is 2, in the age group of 35–44, the observed sex ratio is 3, and afterwards the sex ratio linearly decreases with age. Across life, the mean sex ratio is evaluated at 2 (Klaeboe et al., 2005; Wiemels et al., 2010a).

**Hormonal Influence During Pregnancy**: The incidence of meningioma in women at the age of fertility is low and meningiomas rarely develop over the course of pregnancy (prevalence of 0.1–0.5%). However, during pregnancy an increased meningioma growth has been observed in some cases and may lead to life-threatening complications. (Dumitrescu et al., 2014; Barghouthi et al., 2020).

Pregnancy-induced meningiomas growth can reverse as these meningioma usually shrink after delivery (Kerschbaumer et al., 2015; Lusis et al., 2012). As symptomatic pregnancy-induced meningioma growth is rare, there are no clinical studies available regarding the relative growth of meningioma during pregnancy.

**Increased Risk in Patients with Breast Cancer**: A 26% increased risk of meningiomas has been observed in patients with breast cancer, regardless of the treatment these patients received (Lopez-Rivera et al., 2020; Miller, 1986). The association of both tumors with hormone receptors as a cause of this increased risk was disconfirmed by the finding that there was no association between breast cancer hormone receptor status and the risk of developing a meningioma (Lopez-Rivera et al., 2020).

**Increased risk of meningioma in men treated with antiandrogens:** Cyproterone use was associated with increased risk of meningioma in transgender patients (male to female). The incidence of meningioma was found to be 20.7 per 100,000 person years in this cohort (Weill et al., 2021).

These findings raise concerns about the potentially increased risk of meningioma development in women undergoing hormone therapies, including contraceptives and menopause hormonal treatment (MHT). The influence of sex hormones on meningiomas has been recognized for decades, and further research is needed and ongoing to understand the mechanisms and clinical relevance of this association.

### 2.2 Hormone receptors in meningiomas

The presence of estrogen receptors (ER) and progesterone receptors (PR) in meningioma tissue was initially reported in the late 1970s and early 1980s (Miller, 1986). Since these early reports, numerous studies have investigated the expression of progesterone, estrogen and androgen receptors in both normal leptomeninges and meningiomas.

### 2.3 Progesterone receptors (PRs)

The majority of meningiomas exhibit PR expression, with more than 90% reported in a recent study (Portet et al., 2020). The expression of PR is higher in premenopausal women (78.2%) compared to postmenopausal women (68.4%) and men (65.1%) (Agopiantz et al., 2023).

Higher PR expression has been associated with a more favorable prognosis and a lower risk of recurrence (Pravdenkova et al., 2006; Hsu et al., 1997). Some studies have reported a correlation between PR expression and the WHO grade of meningiomas. Grade 1 tumors tend to have higher PR expression, while grade 2 tumors show lower expression. Grade 3 meningiomas often exhibit very low or no PR expression (Roser et al., 2004). However, this finding has not been consistent across all studies (Takei et al., 2007; Korhonen et al., 2006; Abdelzaher et al., 2010). It's worth noting that the variability in defining PR expression through scoring and cutoff values limits the comparability of these findings.

### 2.4 Estrogen receptors (ER)

ER expression has been described in up to 30% of meningiomas, and this expression has been associated with an unfavorable prognosis in some studies (Hua et al., 2017). Estrogen receptors were found in 8.7%, 1.6% and 6.8% of grade 1, 2, and 3 meningiomas, respectively (Agopiantz et al., 2023). However, other investigations have reported low ER expression and found no correlation with recurrence rates (Liu et al., 2018). The expression of ER in women (14.7%) is higher compared to men (7.5%)

### 2.5 Androgen receptors (AR)

The expression of AR in meningiomas varies across studies, with a prevalence of up to 88% in recent publications (Portet et al., 2020; Carroll et al., 1995). Variability in receptor expression can be attributed to improvements in the sensitivity of techniques for detecting RNA or protein expression over time. Therefore, larger studies employing more sensitive tools are needed to better correlate the receptor expression in surgical specimens with the clinical presentation and the outcomes in meningiomas.

This highlights the complex landscape of sex hormone receptor expression in meningiomas and underscores the need for further research to clarify their clinical implications. The variability in study findings and the evolving detection techniques require larger, more comprehensive studies to better understand the role of these receptors in meningioma development and progression. A recent study on a cohort of male patients using anabolic androgenic steroids did not find an increased risk of meningioma development (Giraldi et al., 2024).

### 2.6 Oral contraceptives

Oral contraceptives (OCs) and, especially, combined hormonal contraception (CHCs) are commonly prescribed, and their potential association with the risk of meningiomas has been the subject of various studies with conflicting results. Most of the earlier studies, primarily case-control studies, showed no increased risk of meningiomas in individuals using hormonal therapy (Samarut et al., 2021; Cea-Soriano et al., 2011; Hatch et al., 2005b; Custer et al., 2006b; Lee et al., 2006; Claus et al., 2012; Korhonen et al., 2010). The Nurses' Health Study (NHS) (Jhawar et al., 2003) reported that the use of OCs, both current and past, was not statistically significantly associated with an increased risk of meningiomas when compared to nonusers. The Iowa Women's Health Study (Johnson et al., 2011) also found no increase in the risk of meningiomas among women who had ever used OCs. A recent meta-analysis (Yang et al., 2020), which included data from 13 studies, did not find conclusive evidence of an association between OC use and the risk of meningioma in female patients. The relative risk (RR) was reported as 0.99 (95% CI, 0.87–1.13; I2 = 42.7%).

In 2006, a study by Lee et al. (2006) suggested a potential protective role of OCs in premenopausal women, particularly in current users.

Two retrospective studies reported a modestly increased risk of meningioma with OCs: OR = 1.5, 95% CI 0.8–2.7 (Hatch et al., 2005a; Custer et al., 2006a). Only one prospective study by Michaud et al. (2010) reported an increased risk of meningiomas in current users of hormonal contraceptives. The hazard ratio (HR) was 3.61 (95% CI, 1.75–7.46) when compared to women who had never used OCs. The potential for diagnostic bias was noted in this study, as women using OCs might receive more frequent medical surveillance and follow-up when experiencing symptoms that could lead to the diagnosis of meningioma.

The discrepancies in the results of these studies may be attributed in part to variations in the type of OC use, the duration of OC treatment, and whether hormonal treatment was current or past. Noteworthy, a myriad of OC products are available, with doses that have diminished over time and the development of the minipill, Progestin-Only Pill (POP), as well as different combinations of estrogens and progestins. Some products are no longer commercialized. Additionally, differences in study design and potential biases stemming from self-reported hormone use versus prescription data might also contribute to these inconsistencies. Therefore, in case of meningioma, a thorough analysis of past gynecological conditions and previous OC prescriptions should be considered.

### 2.7 Progestins

Progestins, synthetic compounds emulating the action of progesterone, serve a pivotal role in various medical applications, spanning from contraception to postmenopausal hormone replacement therapy. These medications interact with progesterone receptors, exhibiting distinct effects based on their generational classification or structural composition (Edwards and Can, 2024).

Progestins are used in both sexes although more specifically in women. They are used in several conditions and symptoms. They can regulate the menstrual cycle and treat abnormal uterine bleeding, prevent abortion and preterm birth, treat symptoms associated to endometriosis, prevent endometrial proliferation or help treat certain cancers. In adolescents and women of reproductive age, progestins are found in hormonal contraceptives. In postmenopausal women with an intact uterus, progestins are part of the menopause hormonal treatment.

Several Progestin agonists have to date been associated to meningioma: Cyproterone Acetate (CPA), Nomegestrol Acetate (NA), Chlormadinone Acetate (CMA), Promegestone, medrogestone, and medroxyprogesterone.

CPA is a synthetic progestin derived from hydroxyprogesterone, exhibiting antagonist properties on AR. While not approved for use in the United States, it is prescribed in several European countries and Canada for various indications, such as hirsutism or acne (idiopathic or in the context of hyperandrogenism due to different conditions such as polycystic ovary syndrome or adrenal hyperplasia), alone or combined with ethinylestradiol (EE). The indications and dosages for CPA vary among countries, as outlined by Weill et al. (2021).

In male patients, CPA is employed in the palliative treatment of prostate cancer and hypersexuality disorder. It is also used in male-to-female (MTF) gender-affirming hormone therapy in conjunction with estrogen therapy, as it acts centrally and suppresses gonadotropins, thereby reducing testosterone production (Prince and Safer, 2020).

The initial suspicion of Progestin-associated meningioma was a CPA-associated meningioma and emerged in a transgender MTF patient, diagnosed with a meningioma of the olfactory groove after five years of treatment with 100 mg of CPA daily in addition to estrogen treatment (Gazzeri et al., 2007). Subsequently, case reports have been published regarding meningiomas in both men and women treated with daily doses of CPA ranging from 25 to 100 mg over periods of 4–30 years.

Data from the EPI-PHARE study, a national epidemiological investigation conducted in France on a large cohort (1,300,000 person-years exposed) revealed that the incidence of meningiomas requiring surgery or radiotherapy in the group exposed to a high cumulative dose of CPA (defined as a cumulative dose of at least 3 g during the first 6 months) was significantly higher than in the control group. The study demonstrated a clear, strong, dose-dependent relationship between CPA use and the risk of meningiomas requiring invasive treatment. A dose-dependent association between CPA and meningiomas requiring treatment by surgery or radiotherapy has been demonstrated and increased CPA doses elevate the risk of having multiple meningioma surgeries and meningioma occurring at multiple locations. (Weill et al., 2021). Notably, this study was conducted in a cohort of patients treated between 2007 and 2014. During these times, the association between CPA and meningioma was unknown, moreover, it was unknown that cessation of CPA would preclude the need for surgery or radiation therapy in 98.1% of cases (Malaizé et al., 2021). Therefore, we can state that CPA increases the risk of meningioma, but not necessarily the risk of surgery and/or radiation therapy to treat meningioma. Other studies with smaller cohorts reported the same results. A case cohort study from the United Kingdom of 2849 male patients receiving hormonal therapy reported an increased risk of meningiomas among male current users of high-dose CPA (50 mg/day) with an odds ratio (OR) of 6.30 compared with nonusers. No statistically significant association was observed in women who received a relatively low dose of 2 mg/day of CPA in conjunction with estrogen (Cea-Soriano et al., 2011). A retrospective population-based cohort study in Spain by Gil et al. (2011), identified two men and two women with meningiomas among 2474 CPA users (median dose of 50 mg/day, duration >1 year). Patients with a history or current use of CPA present with significantly more meningiomas and are diagnosed at a younger age compared to patients without hormonal exposure (Samarut et al., 2021).

There is a growing body of evidence linking other progestin use, particularly NA and CMA, to the risk of meningiomas (Hoisnard et al., 2022). The next EPI-PHARE study published in 2023 examined data from 18,000 women who had undergone meningioma surgery and 90,000 control women between 2009 and 2018. Promegestone (Surgestone 0.5 mg), medrogestone (Colprone 5 mg), and medroxyprogesterone acetate (Depo Provera 150 mg/3 ml) showed a correlation between prolonged use and increased meningioma risk. The risk ratios were 2.7, 4.1, and 5.6, respectively. Meningiomas before the age of 45 years were rare in cases of exposure to medrogestone (n = 3/42), medroxyprogesterone acetate (n = 3/9), or promegestone (n = 10/83), and only one (medroxyprogesterone) was observed before the age of 35. Importantly, this risk increased when the duration of usage exceeded one year. In this study, three quarters of women were exposed for more than one year.

In contrast, in this EPI-PHARE 2023 study, intrauterine devices containing levonorgestrel, with dosages of 13.5 and 52 mg, did not exhibit an elevated risk of meningioma. Moreover, this study was exploring other hormonal medications such as micronized progesterone (oral, intravaginal, and cutaneous) (Utrogestan and generics) and dydrogesterone (Duphaston, Climaston, Femoston) and showed that exposure to these substances was not significantly associated with an increased risk of intracranial meningioma surgery (Roland et al., 2023). For other progestins used alone, as dienogest, drospirenone, desogestrel the risk in not known.

A unique feature of Progestin-associated Meningioma is regression or stabilization upon treatment discontinuation. In a recent French retrospective study involving 71 adult women with 125 progestin-associated meningiomas, regression was observed in 29.6% of cases, stability in 68.5%, and continued growth in 1.9% of cases (Malaizé et al., 2021; Voormolen et al., 2021), whereas the natural course of meningioma annual growth rate, depending on the age and T2 MRI appearance, was estimated by Yamada between 3.4% in the low-risk group (low T2 signal), 8.2% in the intermediate risk group (>50 years, high T2 signal) and 17.5% for the high-risk (<50 years, high T2 signal) group (Yamada et al., 2023). CPA has since been associated with several reports of regression or stabilization of meningiomas upon treatment withdrawal (Bernat et al., 2015, 2018; Cebula et al., 2010; Kalamarides and Peyre, 2017; Gonçalves et al., 2010). There are also reports of meningiomas that regress in size after cessation of CMA and NA treatment (Passeri et al., 2019; Devalckeneer et al., 2021).

Another feature of Progestin-associated Meningiomas is their location. They appear to be primarily located at the skull base, in the anterior and middle cranial fossae, in contact with the body and the wings of the sphenoid bone (Graillon et al., 2021).

Additionally, Progestin-associated meningiomas seem to exhibit a higher frequency of somatic *PIK3CA* mutations, suggesting a hormone-induced mutational shift promoting growth and increasing cell invasion (Abedalthagafi et al., 2016).

Compared to non-Progestin induced intracranial meningiomas and spinal meningiomas, Progestin-associated meningiomas were found to be the most progesterone receptor (PR) positive (97% positivity) and had the strongest receptor-positive immunoreactivity in a study determining the receptor status by tissue microarray and immunohistochemistry (Portet et al., 2020).

The causal link between Progestin use and the risk of meningioma was first doubted but is now acknowledged because of its specific features: the dose-effect relationship, the reduction of risk after treatment discontinuation, the specificity of the tumor location, and the specificity of the somatic mutational landscape of Progestin-associated meningiomas.

In Belgium, CPA is registered under the name Androcur and is included in the combined oral contraceptive (COC) under the name Diane(-35), NA under the tradename of Lutenyl, Nogest, Nomegestrol Stragen and included in the COC under the name Zoely. Currently no case of Zoely-induced meningioma has been reported. Considering CMA, Luteran 5mg and 10 mg are currently withdrawn from the market in Belgium. CMA is included in CHC at 2mg, commercialized under the names Bellina or Helen.

CPA, NA, CA and dydrogesterone are currently contraindicated in France and in Belgium for use in patients with a history of meningioma, and are under review by the European Medicines Agency. Progestins in general are never recommended in case of history of meningioma. The European Medicines Agency has recently imposed restrictions on the use of CPA in doses exceeding 10 mg/day due to the risk of meningiomas.

Stopping progestins is not always an option as mechanical contraception such as copper IUD may often worsen the blood flow and/or dysmenorrhea. Progestins also have non-contraceptive indications in the treatment of gynecological conditions. Among these, endometriosis, menorrhagia, dysmenorrhea, or myomas are frequent reasons for gynecological referral. These pathologies often occur in young patients, with a future desire for pregnancy and for which surgery as hysterectomy, endometrectomy for menorrhagia or tubal ligation, is not an option as these would irremediably compromise their fertility.

The progestin withdrawal must thus be weighed against the natural evolution of gynecological conditions. Pros and cons of maintaining progestin treatment must be explained to the patients. Multidisciplinary meetings including skull base surgeons, gynecologists, endocrinologists discussing hormonal and surgical options depending on the age, the desire for future pregnancies and the symptoms of the patient are advised in meningioma cases.

### 2.8 Menopause Hormonal Treatment

The association between the use of MHT and the risk of meningioma has yielded inconclusive results. Some studies have shown an increased risk (Wigertz et al., 2006; Blitshteyn et al., 2008; Andersen et al., 2013; Jhawar et al., 2003; Benson et al., 2010; Michaud et al., 2010; Korhonen et al., 2012), while others have not (Cea-Soriano et al., 2011; Hatch et al., 2005b; Custer et al., 2006b; Lee et al., 2006; Claus et al., 2012; Johnson et al., 2011).

Concerns regarding meningiomas and MHT were initially raised following the Nurses' Health Study (NHS), which reported a relative risk (RR) for meningiomas of 1.86 (95% CI, 1.07–3.24) in postmenopausal women currently using MHT compared to nonusers (Jhawar et al., 2003). Notably, this increased risk was not observed in past users of MHT, with a RR of 1.01 (95% CI, 0.49–2.10). In contrast, a study by Wigertz et al. (2006) reported an increased risk of meningioma in postmenopausal women who had ever used MHT, with an odds ratio (OR) of 1.7 (95% CI, 1.0–2.8).

The Million Women Study also observed a risk of 1.34 (95% CI, 1.03–1.75) for meningiomas in current MHT users compared to postmenopausal women who had never received MHT (Benson et al., 2010). This study yielded significantly increased risks for meningiomas in users of estrogen-only therapy (1.31 (1.20–1.43)), but not estrogen-progestin therapy (1.05 (0.95–1.16)) (Green et al., 2019; Benson et al., 2014).

A recent case-control study in China revealed that prior use of MHT was associated with an increased risk of meningioma, with an odds ratio of 1.2 (95% CI, 1.0–1.4). Those who received combination therapy with estrogens and progestogens had a more prominent risk (OR of 1.3, 95% CI, 1.1–1.6) compared to progestin-only or estrogen-only users. This study included indications for hormone use such as menopausal treatment and symptom alleviation for various gynecological conditions (Shu et al., 2019).

In contrast, several studies failed to demonstrate an increased risk of meningiomas in postmenopausal patients on MHT (Cea-Soriano et al., 2011; Hatch et al., 2005b; Custer et al., 2006b; Lee et al., 2006; Claus et al., 2012; Johnson et al., 2011).

Meta-analyses have provided mixed results. One meta-analysis of 14 studies reported a relative risk of 1.19 (95% CI, 1.01–1.40) (Qi et al., 2013), while another meta-analysis of 11 studies found an odds ratio of 1.29 (95% CI, 1.03–1.60) (Fan et al., 2013). A large prospective study by Benson et al., in 2010 (Benson et al., 2010) described an increased risk of meningiomas in postmenopausal estrogen and estrogen-progestagen users. A subsequent meta-analysis (Benson et al., 2014) combining their results with other published studies confirmed an increased risk of meningioma with a RR of 1.35 (95% CI, 1.21–1.49). This risk was statistically significant for estrogen-only but not for combined estrogen-progestin treatment. Up to now micronized progesterone is not incriminated in meningioma.

Regarding the effect of MHT on the growth rate of meningiomas, one recent study analyzed the growth rate in patients receiving estrogen therapy and found a lower growth rate compared to age-matched controls not on estrogen. This suggests that estrogen replacement therapy may be safe in patients with meningiomas (Dresser et al., 2020).

This highlights the variability in study results concerning the association between MHT and meningiomas, underlining the need for ongoing research and a personalized approach to hormone therapy in postmenopausal women. Healthcare providers should consider the available evidence, individual patient risk factors, and patient preferences when making treatment decisions regarding MHT.

### 2.9 Fertility treatment and meningioma: limited data

The association between fertility treatment and meningioma remains an area with limited available data.

There have been two case reports describing the diagnosis of meningioma in women with a history of fertility treatment. Additionally, one case documented the rapid growth of a meningioma detected one year after unsuccessful fertility treatment (Patterson and Elashaal, 2016).

Data from the study conducted by Korhonen et al. did not reveal an increased risk of meningiomas in women with a history of fertility treatment. However, it's important to note that only 45 patients had received fertility treatment in their patient population (Korhonen et al., 2006).

In a retrospective review involving 206 patients with meningiomas, the group of 26 female patients who had undergone fertility treatment presented at a younger age and were more likely to have multiple meningiomas compared to the group with no history of fertility treatment (Shahin et al., 2018).

These findings suggest that future studies are needed to study the effect of hormonal stimulation for assisted-reproductive technologies on meningiomas. Currently, the available data are too limited to provide valid recommendations.

### 2.10 Gender-affirming therapies

Transgender women who receive CPA may face an elevated risk of development or growth of intracranial meningiomas, especially given the high doses of CPA used (100 mg) in this population. When dealing with presumed CPA-associated meningioma, discontinuing the medication seems to be a suitable management strategy, particularly when surgery is not urgently required to address elevated intracranial pressure or prevent neurological deterioration (Millward et al., 2021, 2022).

Considering the significance of gender-affirming hormone therapy for transgender individuals, it is crucial to offer a suitable alternative hormone regimen and in many countries, spironolactone is currently prescribed as a first-line androgen-lowering therapy to avoid the risks associated with CPA use (Sehgal, 2023). The use of CPA in both high doses and for extended periods is currently declining in this population (Millward et al., 2022).

### 2.11 Conclusions and general guidelines Fig. 1

#### 2.11.1 Withdrawal

In case of meningioma, all preparations containing progestin must be stopped. Studies have shown regression or stabilization of meningiomas upon discontinuation of progestin treatment. Discontinuing progestin is an appropriate management strategy, especially when surgery is not urgently required.

**Fig. 1:**
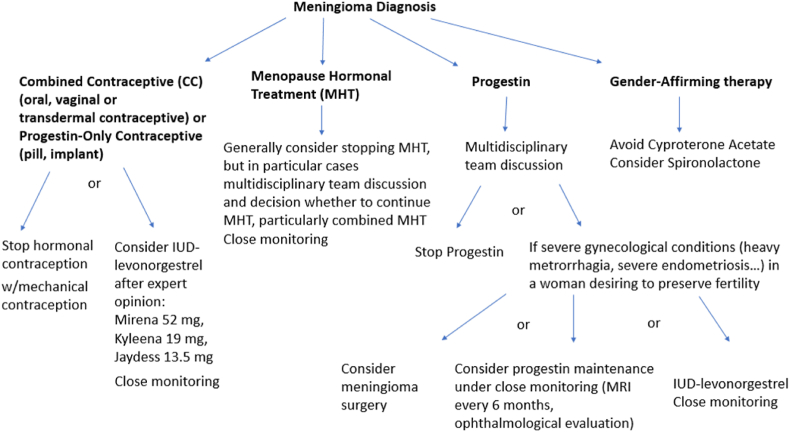
Decision tree proposal regarding sex hormone treatment after Meningioma Diagnosis.

#### 2.11.2 Oral contraception

All hormonal contraceptives contain progestin and are based on progestin properties. The use of combined hormonal contraception (CHCs) is not consistently associated with an increased risk of meningioma, but if a meningioma is diagnosed, cessation of hormonal contraception should be considered. In case of higher risk of meningioma: personal and/or familial history of meningioma, history of cranial ionizing radiation, avoiding progestins is advised on a personal basis.

**Cyproterone Acetate (**CPA**) and Other Progestins**: Evidence strongly supports an association between CPA use and the development of meningiomas. Other progestin treatments, including CMA, NA and medroxyprogesterone, have also shown a higher risk. As a pharmacologic class effect is suspected, the use of progestins in a patient suffering from a meningioma should be avoided. Very close clinical surveillance is advised for patients on these treatments, especially in case of risk factors of meningiomas or in older patients with high daily doses and long-term exposure. Whenever possible, these treatments should be prescribed in a limited timeframe in patients with no proven meningioma. Healthcare providers should inform patients about the potential risks associated with progestin use, particularly CPA and NA, and the development of meningiomas. Alternative treatment should be proposed.

**Menopause Hormonal Treatment (MHT)**: Although not well established, a moderately increased risk of meningioma has been reported in MH-treated patients. Micronized progesterone does not seem to be associated with an increased meningioma risk.

Until more studies are available to provide scientific data, it is advisable to avoid the use of MHT in women with meningiomas.

Breast cancer risk seems to be increased in case of meningioma. Female patients with meningioma should be screened for breast cancer.

#### 2.11.3 Gender-affirming therapies

Transgender women on high-dose CPA face higher risks of meningioma development and growth. Discontinuing CPA and offering alternative androgen-lowering treatments is advised in any case of meningioma in this population. Currently, spironolactone has become the first-line androgen-lowering therapy in transgender women.

#### 2.11.4 Monitoring

For patients on progestin therapy, healthcare providers should implement a monitoring plan that includes yearly physical exams, especially focusing on neurological and ophthalmological status and imaging studies, particularly for individuals with a higher risk of developing meningiomas. Periodic MR imaging identifies the presence of tumors or tumoral growth and the physical exam detects early symptoms of a meningioma. For patients with risk factors (genetic disorders, personal and/or familial history of meningioma or breast cancer, history of ionizing radiation), an MRI should be obtained before starting any progestin. In the absence of risk factors, at least an MRI should be obtained at five years after CPA, NA or CA treatment and then every two years. An MRI at baseline can be considered but this question should be the subject of a Health Economic analysis.

These recommendations are based on the currently available evidence, but it's essential to note that ongoing research may provide additional insights into the relationship between hormonal treatments and meningiomas. Healthcare providers should stay informed about the latest developments in this field.

In order to warn the national and international medicine agencies, healthcare practitioners should report the cases of meningiomas associated to progestins. In Europe, connecting to the site of the European Medicines Agency (EMA) www.ema.europa.eu/en, one can find the list of national registers where such side effects can be reported.

## Funding and conflicts of interests

The authors did not receive any specific funding for this research. The authors have no conflicts of interest to disclose.

## Declaration of competing interests

All authors declare they have no competing interests.

## References

[bib1] Abdelzaher E., El-Gendi S., Yehya A., Gowil A.G. (2010). Recurrence of benign meningiomas: predictive value of proliferative index, BCL2, p53, hormonal receptors and HER2 expression. Br. J. Neurosurg..

[bib2] Abedalthagafi M., Bi W.L., Aizer A.A., Merrill P.H., Brewster R., Agarwalla P.K., Listewnik M.L., Dias-Santagata D., Thorner A.R., Van Hummelen P., Brastianos P.K., Reardon D.A., Wen P.Y., Al-Mefty O., Ramkissoon S., Folkerth R.D., Ligon K.L., Ligon A.H., Alexander B.M., Dunn I.F., Beroukhim R., Santagata S. (2016). Oncogenic PI3K mutations are as common as AKT1 and SMO mutations in meningioma. Neuro Oncol..

[bib3] Agopiantz M., Carnot M., Denis C., Martin E., Gauchotte G. (2023). Hormone receptor expression in meningiomas: a systematic review. Cancers.

[bib4] Andersen L., Friis S., Hallas J., Ravn P., Schrøder H.D., Gaist D. (2013). Hormone replacement therapy increases the risk of cranial meningioma. European journal of cancer (Oxford, England : 1990).

[bib5] Baldi I., Gruber A., A A., Berteaud E., P L., Huchet A., Tourdias T., Kantor G., Maire J.P., Vital A., Loiseau H. (2011). Descriptive epidemiology of CNS tumors in France: results from the Gironde Registry for the period 2000–2007. Neuro Oncol..

[bib6] Banerjee J., Pääkkö E., Harila M., Herva R., Tuominen J., Koivula A., Lanning M., Harila-Saari A. (2009). Radiation-induced meningiomas: a shadow in the success story of childhood leukemia. Neuro Oncol..

[bib7] Barghouthi T., Lemley R., Figurelle M., Bushnell C., Steegers E.A.P., Cipolla M.J., Miller E.C. (2020).

[bib8] Benson V.S., Kirichek O., Beral V., Green J. (2014). Menopausal hormone therapy and central nervous system tumor risk: large UK prospective study and meta-analysis. Int. J. Cancer.

[bib9] Benson V.S., Pirie K., Green J., Bull D., Casabonne D., Reeves G.K., Beral V. (2010). Hormone replacement therapy and incidence of central nervous system tumours in the Million Women Study. Int. J. Cancer.

[bib10] Bernat A.L., Bonnin S., Labidi M., Aldahak N., Bresson D., Bouazza S., Froelich S. (2018). Regression of giant olfactory groove meningioma and complete visual acuity recovery after discontinuation of cyproterone acetate. J. Ophthalmic Vis. Res..

[bib11] Bernat A.L., Oyama K., Hamdi S., Mandonnet E., Vexiau D., Pocard M., George B., Froelich S. (2015). Growth stabilization and regression of meningiomas after discontinuation of cyproterone acetate: a case series of 12 patients. Acta Neurochir..

[bib12] Blitshteyn S., Crook J.E., Jaeckle K.A. (2008). Is there an association between meningioma and hormone replacement therapy. J. Clin. Oncol. : official journal of the American Society of Clinical Oncology.

[bib13] Caroli E., Salvati M., Giangaspero F., Ferrante L., Santoro A. (2006). Intrameningioma metastasis as first clinical manifestation of occult primary breast carcinoma. Neurosurg. Rev..

[bib14] Carroll R.S., Zhang J., Dashner K., Sar M., Wilson E.M., Black P.M. (1995). Androgen receptor expression in meningiomas. J. Neurosurg..

[bib15] Cea-Soriano L., Blenk T., Wallander M.-A., Rodríguez L.A.G. (2011). Hormonal therapies and meningioma: is there a link?. Cancer epidemiology.

[bib16] Cebula H., Pham T.Q., Boyer P., Froelich S. (2010). Regression of meningiomas after discontinuation of cyproterone acetate in a transsexual patient. Acta Neurochir..

[bib17] Claus E.B., Calvocoressi L., Bondy M.L., Schildkraut J.M., Wiemels J.L., Wrensch M. (2011). Family and personal medical history and risk of meningioma. J Neurosurg.

[bib18] Claus E.B., Calvocoressi L., Bondy M.L., Wrensch M., Wiemels J.L., Schildkraut J.M. (2012). Exogenous hormone use, reproductive factors, and risk of intracranial meningioma in females. J. Neurosurg..

[bib19] Cushing H. (1929). Meningiomas arising from the tuberculum sellae with the syndrome of primary optic atrophy and bitemporal field defects combined with a normal sella turcica in a middle-aged person. Acta Neuropathol..

[bib20] Custer B., Longstreth W.T., Phillips L.E., Koepsell T.D., Van Belle G. (2006). Hormonal exposures and the risk of intracranial meningioma in women: a population-based case-control study. BMC Cancer.

[bib21] Custer B., Longstreth W.T., Phillips L.E., Koepsell T.D., van Belle G. (2006). Hormonal exposures and the risk of intracranial meningioma in women: a population-based case-control study. BMC Cancer.

[bib22] Degeneffe A., De Maertelaer V., De Witte O., Lefranc F. (2023). The association between meningioma and breast cancer: a systematic review and meta-analysis. JAMA Netw. Open.

[bib23] Devalckeneer A., Aboukais R., Bourgeois P., De Witte O., Racape J., Caron S., Perbet R., Maurage C.A., Lejeune J.P. (2021). Preliminary report of patients with meningiomas exposed to Cyproterone Acetate, Nomegestrol Acetate and Chlormadinone Acetate - monocentric ongoing study on progestin related meningiomas. Clin. Neurol. Neurosurg..

[bib24] Dolecek T.A., Dressler E.V., Thakkar J.P., Liu M., Al-Qaisi A., Villano J.L. (2015). Epidemiology of meningiomas post-public law 107-206: the benign brain tumor cancer registries amendment act. Cancer.

[bib25] Dresser L., Yuen C., Wilmington A., Walker M.T., Vogel T.J., Merrell R., Kamson D.O. (2020). Estrogen hormone replacement therapy in incidental intracranial meningioma: a growth-rate analysis. Sci. Rep..

[bib26] Dumitrescu B.C., Tataranu L.G., Gorgan M.R. (2014). Pregnant woman with an intracranial meningioma: case report and review of the literature. Romanian Neurosurgery.

[bib27] Edwards M., Can A.S. (2024). StatPearls. StatPearls Publishing Copyright © 2024, StatPearls Publishing LLC., Treasure Island (FL) Ineligible Companies. Disclosure: Ahmet Can Declares No Relevant Financial Relationships with Ineligible Companies.

[bib28] Fan Z., Shen J., Wu Y., Yu H., Zhu Y., Zhan R. (2013). Hormone replacement therapy and risk of meningioma in women: a meta-analysis. Cancer causes & control : CCC (Cancer Causes Control).

[bib29] Francois P., N'Dri D., Bergemer-Fouquet A.M., Ben Ismail M., Papagiannaki C., Cottier J.P., Jan M. (2010). Post-traumatic meningioma: three case reports of this rare condition and a review of the literature. Acta Neurochir..

[bib30] Gazzeri R., Galarza M., Gazzeri G. (2007). Growth of a meningioma in a transsexual patient after estrogen-progestin therapy. N. Engl. J. Med..

[bib31] Gil M., Oliva B., Timoner J., Maciá M.A., Bryant V., de Abajo F.J. (2011). Risk of meningioma among users of high doses of cyproterone acetate as compared with the general population: evidence from a population-based cohort study. Br. J. Clin. Pharmacol..

[bib32] Giraldi L., Heerfordt I.M., Windfeld-Mathiasen J., Dalhoff K.P., Andersen J.T., Horwitz H. (2024). Extensive androgen exposure and meningioma risk - a matched cohort study. Clin. Neurol. Neurosurg..

[bib33] Gonçalves A., Page P., Domigo V., Meder J.F., Oppenheim C. (2010). Abrupt regression of a meningioma after discontinuation of cyproterone treatment. AJNR American journal of neuroradiology.

[bib34] Graillon T., Boissonneau S., Appay R., Boucekine M., Peyrière H., Meyer M., Farah K., Albarel F., Morange I., Castinetti F., Brue T., Fuentes S., Figarella-Branger D., Cuny T., Dufour H. (2021). Meningiomas in patients with long-term exposition to progestins: characteristics and outcome. Neurochirurgie.

[bib35] Green J., Reeves G.K., Floud S., Barnes I., Cairns B.J., Gathani T., Pirie K., Sweetland S., Yang T.O., Beral V. (2019). Cohort profile: the million women study. Int. J. Epidemiol..

[bib36] Hage M., Plesa O., Lemaire I., Raffin Sanson M.L. (2021). Estrogen and progesterone therapy and meningiomas. Endocrinology.

[bib37] Hatch E.E., Linet M.S., Zhang J., Fine H.A., Shapiro W.R., Selker R.G., Black P.M., Inskip P.D. (2005). Reproductive and hormonal factors and risk of brain tumors in adult females. Int. J. Cancer.

[bib38] Hatch E.E., Linet M.S., Zhang J., Fine H.A., Shapiro W.R., Selker R.G., Black P.M., Inskip P.D. (2005). Reproductive and hormonal factors and risk of brain tumors in adult females. Int. J. Cancer.

[bib39] Hoisnard L., Laanani M., Passeri T., Duranteau L., Coste J., Zureik M., Froelich S., Weill A. (2022). Risk of intracranial meningioma with three potent progestogens: a population-based case-control study. Eur. J. Neurol..

[bib40] Hsu D.W., Efird J.T., Whyte Et Hedley (1997). Progesterone and estrogen receptors in meningiomas: prognostic considerations. J. Neurosurg..

[bib41] Hua L., Zhu H., Li J., Tang H., Kuang D., Wang Y., Tang F., Chen X., Zhou L., Xie Q., Gong Y. (2017). Prognostic value of estrogen receptor in WHO Grade III meningioma: a long-term follow-up study from a single institution. J. Neurosurg..

[bib42] Jhawar B.S., Fuchs C.S., Colditz G.A., Stampfer M.J. (2003). Sex steroid hormone exposures and risk for meningioma. J. Neurosurg..

[bib43] Johnson D.R., Olson J.E., Vierkant R.A., Hammack J.E., Wang A.H., Folsom A.R., Virnig B.A., Cerhan J.R. (2011). Risk factors for meningioma in postmenopausal women: results from the Iowa Women's Health Study. Neuro Oncol..

[bib44] Kalamarides M., Peyre M. (2017). Dramatic shrinkage with reduced vascularization of large meningiomas after cessation of progestin treatment. World Neurosurg.

[bib45] Kerr K., Qualmann K., Esquenazi Y., Hagan J., Kim D.H. (2018). Familial syndromes involving meningiomas provide mechanistic insight into sporadic disease. Neurosurgery.

[bib46] Kerschbaumer J., Freyschlag C.F., Stockhammer G., Taucher S., Maier H., Thomé C., Seiz-Rosenhagen M. (2015). Hormone-dependent shrinkage of a sphenoid wing meningioma after pregnancy: case report. J. Neurosurg..

[bib47] Khazanchi R., Nandoliya K.R., Shahin M.N., Rae A.I., Chaliparambil R.K., Bowden S.G., Alwakeal A., Lopez Ramos C.G., Stedelin B., Youngblood M.W., Chandler J.P., Lukas R.V., Sanusi O.R., Dogan A., Wood M.D., Han S.J., Magill S.T. (2024). Obesity and meningioma: a US population-based study paired with analysis of a multi-institutional cohort. J. Neurosurg..

[bib48] Khazanchi R., Nandoliya K.R., Shahin M.N., Rae A.I., Chaliparambil R.K., Bowden S.G., Alwakeal A., Lopez Ramos C.G., Stedelin B., Youngblood M.W., Chandler J.P., Lukas R.V., Sanusi O.R., Dogan A., Wood M.D., Han S.J., Magill S.T. (2024). Obesity and meningioma: a US population-based study paired with analysis of a multi-institutional cohort. J Neurosurg.

[bib49] Klaeboe L., Lönn S., Scheie D., Auvinen A., Christensen H.C., Feychting M., Johansen C., Salminen T., Tynes T. (2005). Incidence of intracranial meningiomas in Denmark, Finland, Norway and Sweden, 1968–1997. Int. J. Cancer.

[bib50] Korhonen K., Auvinen A., Lyytinen H., Ylikorkala O., Pukkala E. (2012). A nationwide cohort study on the incidence of meningioma in women using postmenopausal hormone therapy in Finland. Am. J. Epidemiol..

[bib51] Korhonen K., Raitanen J., Isola J., Haapasalo H., Salminen T., Auvinen A. (2010). Exogenous sex hormone use and risk of meningioma: a population-based case-control study in Finland. Cancer causes & control : CCC (Cancer Causes Control).

[bib52] Korhonen K., Salminen T., Raitanen J., Auvinen A., Isola J., Haapasalo H. (2006). Female predominance in meningiomas can not be explained by differences in progesterone, estrogen, or androgen receptor expression. Journal of neuro-oncology.

[bib53] Lee A., Wallace C., Rewcastle B., Sutherland G. (1998). Metastases to meningioma. AJNR Am J Neuroradiol.

[bib54] Lee E., Grutsch J., Persky V., Glick R.P., Mendes J., Davis F.G. (2006). Association of meningioma with reproductive factors. Int. J. Cancer.

[bib55] Liu F., Wei C., Chen J. (2018). Letter to the Editor. Is there any relationship between estrogen receptor/progesterone receptor status and recurrence of meningioma?. J. Neurosurg..

[bib56] Lopez-Rivera V., Zhu P., Dono A., Lee S., Chen P.R., Ballester L.Y., Sheth S.A., Esquenazi Y. (2020). Increased risk of subsequent meningioma among women with malignant breast cancer. World Neurosurg.

[bib57] Louis D.N., Perry A., Reifenberger G., von Deimling A., Figarella-Branger D., Cavenee W.K., Ohgaki H., Wiestler O.D., Kleihues P., Ellison D.W. (2016). The 2016 world Health organization classification of tumors of the central nervous system: a summary. Acta Neuropathol..

[bib58] Lusis E.A., Scheithauer B.W., Yachnis A.T., Fischer B.R., Chicoine M.R., Paulus W., Perry A. (2012). Meningiomas in pregnancy: a clinicopathologic study of 17 cases. Neurosurgery.

[bib59] Malaizé H., Samoyeau T., Zanello M., Roux A., Benzakoun J., Peeters S., Zah-Bi G., Edjlali M., Tauziède-Espariat A., Dezamis E., Parraga E., Chrétien F., Varlet P., Plu-Bureau G., Oppenheim C., Pallud J. (2021). Evolution of the neurosurgical management of progestin-associated meningiomas: a 23-year single-center experience. Journal of neuro-oncology.

[bib60] Michaud D.S., Gallo V., Schlehofer B., Tjønneland A., Olsen A., Overvad K., Dahm C.C., Kaaks R., Lukanova A., Boeing H., Schütze M., Trichopoulou A., Bamia C., Kyrozis A., Sacerdote C., Agnoli C., Palli D., Tumino R., Mattiello A., Bueno-de-Mesquita H.B., Ros M.M., Peeters P.H.M., van Gils C.H., Lund E., Bakken K., Gram I.T., Barricarte A., Navarro C., Dorronsoro M., Sánchez M.J., Rodríguez L., Duell E.J., Hallmans G., Melin B., Manjer J., Borgquist S., Khaw K.-T., Wareham N.J., Allen N.E., Tsilidis K.K., Romieu I., Rinaldi S., Vineis P., Riboli E. (2010). Reproductive factors and exogenous hormone use in relation to risk of glioma and meningioma in a large European cohort study. Cancer epidemiology, biomarkers & prevention : a publication of the American Association for Cancer Research, cosponsored by the American Society of Preventive Oncology.

[bib61] Miller R.E. (1986). Breast cancer and meningioma. J. Surg. Oncol..

[bib62] Millward C.P., Keshwara S.M., Islim A.I., Jenkinson M.D., Alalade A.F., Gilkes C.E. (2022). Development and growth of intracranial meningiomas in transgender women taking cyproterone acetate as gender-affirming progestogen therapy: a systematic review. Transgend Health.

[bib63] Millward C.P., Phillips E., Alalade A.F., Gilkes C.E. (2021). Gender-affirming hormone therapy associated with multiple meningiomas and atypical histology in a transgender woman. BMJ Case Rep..

[bib64] Moher D., Liberati A., Tetzlaff J., Altman D.G., Group P. (2009). Preferred reporting items for systematic reviews and meta-analyses: the PRISMA statement. BMJ.

[bib65] Moher D., Liberati A., Tetzlaff J., Altman D.G., Group P. (2010). Preferred reporting items for systematic reviews and meta-analyses: the PRISMA statement. Int. J. Surg..

[bib66] Ostrom Q.T., Gittleman H., Truitt G., Boscia A., Kruchko C., Barnholtz-Sloan J.S. (2018). CBTRUS statistical report: primary brain and other central nervous system tumors diagnosed in the United States in 2011-2015. Neuro Oncol..

[bib67] Passeri T., Champagne P.O., Bernat A.L., Hanakita S., Salle H., Mandonnet E., Froelich S. (2019). Spontaneous regression of meningiomas after interruption of nomegestrol acetate: a series of three patients. Acta Neurochir..

[bib68] Patterson A., Elashaal A. (2016). Fast-growing meningioma in a woman undergoing fertility treatments. Case Reports in Neurological Medicine.

[bib69] Portet S., Banor T., Bousquet J., Simonneau A., Flores M., Ingrand P., Milin S., Karayan-Tapon L., Bataille B. (2020). New insights into expression of hormonal receptors by meningiomas. World Neurosurg.

[bib70] Pravdenkova S., Al-Mefty O., Sawyer J.R., Husain M. (2006). Progesterone and estrogen receptors: opposing prognostic indicators in meningiomas. J. Neurosurg..

[bib71] Prince J.C.J., Safer J.D. (2020). Endocrine treatment of transgender individuals: current guidelines and strategies. Expet Rev. Endocrinol. Metabol..

[bib72] Qi Z., Shao C., Huang Y., Hui G.-Z., Zhou Y.-X., Wang Z. (2013). Reproductive and exogenous hormone factors in relation to risk of meningioma in women: a meta-analysis. PLoS One.

[bib73] Rao G., Giordano S.H., Liu J., McCutcheon I.E. (2009). The association of breast cancer and meningioma in men and women. Neurosurgery.

[bib74] Rogers L., Barani I.J., Chamberlain M.C., Kaley T., McDermott M.W., Raizer J., Schiff D., Weber D.C., Wen P.Y., Vogelbaum M.A. (2015). Meningiomas: knowledge base, treatment outcomes, and uncertainties. A RANO review. J. Neurosurg..

[bib75] Roland N.N.A., Hoisnard L., Zureik M., Weill A. (2023).

[bib76] Roser F., Nakamura M., Bellinzona M., Rosahl S.K., Ostertag H., Samii M. (2004). The prognostic value of progesterone receptor status in meningiomas. J. Clin. Pathol..

[bib77] Samarut E., Lugat A., Amelot A., Scharbarg E., Hadjadj S., Primot C., Loussouarn D., Thillays F., Buffenoir K., Cariou B., Drui D., Roualdes V. (2021). Meningiomas and cyproterone acetate: a retrospective, monocentric cohort of 388 patients treated by surgery or radiotherapy for intracranial meningioma. Journal of neuro-oncology.

[bib78] Sehgal I. (2023). Review of adult gender transition medications: mechanisms, efficacy measures, and pharmacogenomic considerations. Front. Endocrinol..

[bib79] Shah D.S., Sanan A., Morell A.A., Eichberg D.G., Shah A.H., Luther E., Lu V.M., Elarjani T., Higgins D.M.O., Patel N.V., Jagid J.R., Ivan M.E., Komotar R.J. (2022). Traumatic brain injury and subsequent brain tumor development: a systematic review of the literature. Neurosurg. Rev..

[bib80] Shahin M., Magill S.T., Ore C.L.D., Peters P., Viner J., McDermott M.W. (2018). MNGI-09. Fertility treatment and meningioma incidence. Neuro Oncol..

[bib81] Shao C., Bai L.P., Qi Z.Y., Hui G.Z., Wang Z. (2014). Overweight, obesity and meningioma risk: a meta-analysis. PLoS One.

[bib82] Shu X., Jiang Y., Wen T., Lu S., Yao L., Meng F. (2019). Association of hormone replacement therapy with increased risk of meningioma in women: a hospital-based multicenter study with propensity score matching. Asia Pac. J. Clin. Oncol..

[bib83] Takei H., Buckleair L.W., Powell S.Z.-E. (2007). Immunohistochemical expression of apoptosis regulating proteins and sex hormone receptors in meningiomas. Neuropathology : official journal of the Japanese Society of Neuropathology.

[bib84] Turner N., Kaye A.H., Paldor I. (2021). Metastases to meningioma-review and meta-analysis. Acta Neurochir..

[bib85] Umansky F., Shoshan Y., Rosenthal G., Fraifeld S., Spektor S. (2008). Radiation-induced meningioma. Neurosurgical Focus FOC.

[bib86] Vernooij M.W., Ikram M.A., Tanghe H.L., Vincent A.J., Hofman A., Krestin G.P., Niessen W.J., Breteler M.M., van der Lugt A. (2007). Incidental findings on brain MRI in the general population. N. Engl. J. Med..

[bib87] Voormolen E.H.J., Champagne P.O., Roca E., Giammattei L., Passeri T., di Russo P., Sanchez M.M., Bernat A.L., Yoldjian I., Fontanel S., Weill A., Mandonnet E., Froelich S. (2021). Intracranial meningiomas decrease in volume on magnetic resonance imaging after discontinuing progestin. Neurosurgery.

[bib88] Weill A., Nguyen P., Labidi M., Cadier B., Passeri T., Duranteau L., Bernat A.-L., Yoldjian I., Fontanel S., Froelich S., Coste J. (2021). Use of high dose cyproterone acetate and risk of intracranial meningioma in women: cohort study. BMJ (Clinical research ed).

[bib89] Wiemels J., Wrensch M., Claus E.B. (2010). Epidemiology and etiology of meningioma. J. Neuro Oncol..

[bib90] Wiemels J.L., Wrensch M., Claus E.B. (2010). Epidemiology and etiology of meningioma. Journal of neuro-oncology.

[bib91] Wigertz A., Lönn S., Mathiesen T., Ahlbom A., Hall P., Feychting M. (2006). Risk of brain tumors associated with exposure to exogenous female sex hormones. Am. J. Epidemiol..

[bib92] Yamada S., Hirayama R., Iwata T., Kuroda H., Nakagawa T., Takenaka T., Kijima N., Okita Y., Kagawa N., Kishima H. (2023). Growth risk classification and typical growth speed of convexity, parasagittal, and falx meningiomas: a retrospective cohort study. J Neurosurg.

[bib93] Yamanaka R., Hayano A., Kanayama T. (2017). Radiation-induced meningiomas: an exhaustive review of the literature. World Neurosurg.

[bib94] Yang X., Liu F., Zheng J., Cheng W., Zhao C., Di J. (2020). Relationship between oral contraceptives and the risk of gliomas and meningiomas: a dose-response meta-analysis and systematic review. World Neurosurg.

